# Tuberculosis and Increased Incidence of Cardiovascular Disease: Cohort Study Using United States and United Kingdom Health Records

**DOI:** 10.1093/cid/ciae538

**Published:** 2024-11-04

**Authors:** Julia A Critchley, Elizabeth S Limb, Anjali Khakharia, Iain M Carey, Sara C Auld, Stephen De Wilde, Tess Harris, Lawrence S Phillips, Derek G Cook, Mary K Rhee, Umar A R Chaudhry, Liza Bowen, Matthew J Magee

**Affiliations:** Population Health Research Institute, St George's School of Health and ­­Medical Sciences, City St George's, University of London, London, United Kingdom; Population Health Research Institute, St George's School of Health and ­­Medical Sciences, City St George's, University of London, London, United Kingdom; Clinical Studies Center, Atlanta VA Health Care System, Decatur, Georgia, USA; Population Health Research Institute, St George's School of Health and ­­Medical Sciences, City St George's, University of London, London, United Kingdom; Division of Pulmonary, Allergy, Critical Care, and Sleep Medicine, Department of Medicine, Emory School of Medicine, Atlanta, Georgia, USA; Departments of Global Health and Epidemiology, Rollins School of Public Health, Emory University, Atlanta, Georgia, USA; Population Health Research Institute, St George's School of Health and ­­Medical Sciences, City St George's, University of London, London, United Kingdom; Population Health Research Institute, St George's School of Health and ­­Medical Sciences, City St George's, University of London, London, United Kingdom; Clinical Studies Center, Atlanta VA Health Care System, Decatur, Georgia, USA; Division of Endocrinology and Metabolism, Department of Medicine, Emory University School of Medicine, Atlanta, Georgia, USA; Population Health Research Institute, St George's School of Health and ­­Medical Sciences, City St George's, University of London, London, United Kingdom; Clinical Studies Center, Atlanta VA Health Care System, Decatur, Georgia, USA; Division of Endocrinology and Metabolism, Department of Medicine, Emory University School of Medicine, Atlanta, Georgia, USA; Population Health Research Institute, St George's School of Health and ­­Medical Sciences, City St George's, University of London, London, United Kingdom; Population Health Research Institute, St George's School of Health and ­­Medical Sciences, City St George's, University of London, London, United Kingdom; Departments of Global Health and Epidemiology, Rollins School of Public Health, Emory University, Atlanta, Georgia, USA

**Keywords:** tuberculosis, cardiovascular diseases, post-TB, cohort study, electronic health records

## Abstract

**Background:**

Limited evidence suggests elevated risks of cardiovascular disease (CVD) among people diagnosed with tuberculosis (TB) disease, though studies have not adjusted for preexisting CVD risk. We carried out a cohort study using 2 separate datasets, estimating CVD incidence in people with TB versus those without.

**Methods:**

Using data from the United States (Veterans Health Administration) and the United Kingdom (Clinical Practice Research Datalink) for 2000–2020, we matched adults with incident TB disease and no CVD history 2 years before TB diagnosis (US, n = 2121; UK, n = 15 820) with up to 10 people without TB on the basis of age, sex, race/ethnicity and healthcare practice. Participants were followed beginning 2 years before TB diagnosis and for 2 years subsequently. The acute period was defined as 3 months before/after TB diagnosis. TB, CVD, and covariates were identified from electronic routinely collected data (primary and secondary care; mortality). Poisson models estimated incident rate ratios for CVD events in people with TB compared to those without.

**Results:**

CVD incidence was consistently higher in people with TB, including during the baseline period (pre-TB) and particularly in the acute period: incident rate ratios were US, 3.5 (95% confidence interval, 2.7–4.4), and UK, 2.7 (2.2–3.3). Rate ratios remained high after adjusting for differences in preexisting CVD risk: US, 3.2 (2.2–4.4); UK, 1.6 (1.2–2.1).

**Conclusions:**

Increased CVD incidence was observed in people with TB versus those without, especially within months of TB diagnosis, persistent after adjustment for differences in preexisting risk. Enhancing CVD screening and risk management may improve long-term outcomes in people with TB.

## Teaser

We report increased rates of cardiovascular disease (CVD) incidence before and after tuberculosis (TB) diagnosis in a large cohort study. Our findings justify CVD risk assessment during TB care and evaluation of CVD prevention interventions targeting people at elevated TB/CVD risk.

## Graphical Abstract

**Graphical Abstract:**
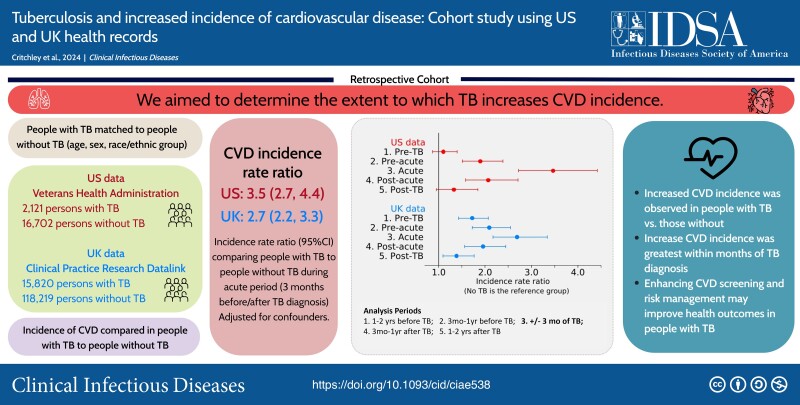
This graphical abstract is also available at Tidbit: https://tidbitapp.io/tidbits/tuberculosis-and-increased-incidence-of-cardiovascular-disease-cohort-study-using-us-and-uk-health-records?utm_campaign=tidbitlinkshare&utm_source=ITP

Globally, tuberculosis (TB) remains a major cause of morbidity, disability and the leading infectious cause of death [1]. In 2021, TB incidence rose to 10.6 million, reversing years of gradual decline before coronavirus disease 2019 (COVID-19) [2, 3]. With an aging global population, TB disease is becoming more common in older people, who have increased risk of multimorbidity, particularly noncommunicable diseases such as cardiovascular disease (CVD) [4]. Antimicrobial treatment for TB disease is highly effective, but TB survivors have a reduced quality of life and higher all-cause mortality [5–9]. TB may itself increase CVD risk because diagnosis can take years [10], resulting in chronic inflammation [11–13], insulin resistance [14, 15], lipid dysregulation [16, 17], endothelial activation, and hypertension [11, 18–22].

Prior studies identified strong associations between TB and CVD. A recent systematic review reported 1.5 times higher risk of major adverse cardiac events among people with a TB history [23], but studies had multiple serious biases including lack of control for confounders, limited sample size, follow-up time, and an inability to observe CVD incidence before TB diagnosis [23].

To our knowledge, no previous studies have assessed the temporal relationship between TB diagnosis and CVD events [23, 24]. However, people diagnosed with TB disease may have an increased risk of CVD even before development of TB symptoms, potentially because of a subacute inflammatory state among those with latent TB infection (LTBI; infection with *Mycobacterium tuberculosis* but no active TB disease), delays in patient healthcare-seeking and health system TB diagnosis, or differences from poor underlying health or access to care. A longitudinal design that allows for observation of CVD incidence before TB diagnosis also provides an opportunity to account for differences in preexisting CVD. Hence, following a cohort before TB diagnosis eliminates most sources of confounding, a potentially serious bias [24]. Our study aimed to determine the extent to which TB increases the risk of incident CVD, even after adjusting for prior differences in CVD incidence during an extended period before the TB diagnosis. We used large retrospective cohorts based on electronic medical records in the United States and United Kingdom; these independent cohorts permitted study design replication and increased statistical power to describe and evaluate the timing of incident CVD events for 2 years before and 2 years after TB diagnosis.

## METHODS

### Study Design

A retrospective matched cohort study in each database (US and UK) separately.

### Data Sources

#### US Data

National veteran population and their healthcare utilization (VHA) datasets were used to construct cohort information including demographics, diagnosis, laboratory measurements, pharmacy prescriptions, and outcomes. US Veterans Eligibility Trends and Statistics dataset (https://rb.gy/ujef83) provided assessment of social determinants related to health.

#### UK Data

The Clinical Practice Research Datalink (CPRD), a large primary care database collects anonymized patient data from a network of General Practices (Primary Care Centers—currently >18 million patients [25]), linked to data sources including Hospital Episode Statistics (data on hospital admissions) [26], Office of National Statistics death registration records, and the Index of Multiple Deprivation (a geospatial measure for socioeconomic status [SES]) [27].

### Study Participants

#### US Data

The sampling frame was all people enrolled in the VHA system aged ≥18 years and having at least 1 primary care visit between 1 January 2000 and 31 December 2019. Those having ≥2 outpatient International Classification of Disease (ICD) codes or ≥1 inpatient ICD code for TB, and a first prescription of pyrazinamide or ethambutol during the same period were considered to have TB disease (see published code lists [28]). The earliest of these dates was defined as TB diagnosis date (index date) [28].

#### UK Data

The sampling frame was all people registered with their CPRD practice at some point between 1 January 2000 and 31 December 2019. People with TB were identified as those aged 18–90 years, with a first record of TB while actively registered in CPRD between these dates using UK primary care diagnostic codes indicative of incident TB [28].

In both datasets, people were required to have no prior record of TB, to be active with their healthcare provider for ≥2 years before the date of TB diagnosis and have no evidence of CVD in primary or secondary records for at least 2 years before the TB diagnosis. Each person with TB was matched with up to 10 people without a TB diagnosis anywhere in their record, creating a match set for each person with TB. For UK people, matching was with replacement, meaning someone without TB could be matched to more than 1 person with TB (Supplementary Text 1). Matching was performed by year of birth, sex, race/ethnicity and either VHA facility (US) or primary care practice (UK). Race (US)/ethnicity (UK) for each person was taken from self-reported information recorded in datasets and classified into broad race/ethnic groups: 3 racial groups for US (White, Black, Mixed/Other) and 4 ethnic groups for UK (White, South Asian, Black, Other). Those with missing race/ethnicity were coded separately (Supplementary Text 1) [29]. People without TB were similarly required to be actively using the healthcare system for at least 2 years before the index date of their matched person with TB and have no prior indication of CVD 2 years before this index date. All people were followed up to the earliest date of: CVD event in primary care or hospital records, death, in the United Kingdom, patient leaving the practice or their practice leaving CPRD, or 1 March 2020. The latter date was chosen because both CVD and TB diagnoses were substantially affected by COVID-19 and lockdowns in 2020 onwards [29].

### Outcomes and Covariates

Date of first CVD diagnosis in primary care or hospital records was identified using ICD-9/ICD-10 codes for US data, primary care diagnostic codes and ICD-10 codes for UK primary care and hospital data respectively [28]. Covariates: information on smoking (ie, never, former, current), body mass index, comorbidities, and prescribing of statins or antihypertensives were extracted at 2 years before TB diagnosis date. SES was measured using quintiles of household income from US Veterans Eligibility Trends and Statistics dataset (https://rb.gy/ujef83) (US) and Index of Multiple Deprivation (UK).

### Statistical Analysis

To estimate CVD incidence over time, we defined a 4-year period comprising ±2 years either side of the TB diagnosis date (index date) divided into 48 separate 30-day periods. For each 30-day period, we searched for an incident CVD event, calculated person-time at risk, and summed these across all match-sets, yielding the rate over each 30-day period for people with and without TB. People who experienced an event were dropped from subsequent 30-day periods. Match sets had to have at least 1 person with and 1 without TB.

Second, we used Poisson models, conditioned on the match sets with an offset for time at risk, and a difference-in-difference analysis to examine risk of an incident CVD event ±2 years of the TB index date. Analyses aggregated time into 5 periods: pre-TB “baseline” (1–2 years before TB index date), pre-acute (3 months to 1 year before TB index date), acute (3 months before and after TB index date), post-acute (3 months to 1 year after TB index date), and post-TB (1–2 years after TB index date). The effect of group (TB vs not TB), time (acute period vs baseline period 1–2 years before TB index date) and the group-time interaction were estimated. Incident rate ratios (IRR) were obtained for people with TB compared to those without for each period. The group-time interaction provided the rate ratio (RR) for the risk of an incident CVD event in people with TB in each period controlling for any differences present in risk during the pre-TB baseline period. This also inherently adjusts for confounding because of personal characteristics as people were their own controls over time [30] (Supplementary Text 1). To add robustness, models were additionally adjusted for HIV status, smoking, body mass index, SES, hypertension, and prescribing of CVD preventive medicines (antihypertensives/statins). Stratified analyses considered effects by age group, sex, and race/ethnicity. Sensitivity analyses included further adjustment for diabetes, limiting to CVD events resulting in hospitalization only (excluding those only diagnosed in primary care), limiting to people with TB who were matched to ≥4 matched people without TB, including immediately fatal CVD events only appearing in linked mortality data and extending post-TB follow-up to 3 years.

All analyses were conducted separately in each dataset using harmonized coding in STATA (example code in Supplementary Text 2).

Ethical approval was granted by the institutional review board at Emory University/Atlanta VHA Medical Center and CPRD's Research Data Governance (protocol 20_000201).

## RESULTS

The final datasets included 17 941 people with TB (US, 2121; UK, 15 820) and 130 494 total matched people without TB (US, 16 702; UK, 113 792 individual people, 118 218 total people without TB) (Supplementary Figure 1*A* and 1*B*). The majority of people with TB had at least 4 matched people without (US, 2034 [96%]; UK, 14 869 [94%]).

People with TB were almost all male in the United States (97% US, 51% UK) and older; median age at diagnosis was 59 years in the United States and 44 years in the United Kingdom (Table 1). Of those with known race/ethnicity, TB diagnoses were predominately in White (54%) and Black (39%) populations in the United States, compared with 32% and 16% respectively for the United Kingdom, with a further 31% of UK diagnoses occurring in South Asian populations. There was a higher proportion of mixed/other ethnicity and missing ethnicity in UK data. A total of 64% of UK people with TB were in the 40% of most deprived areas with similar patterns for those without TB. US data on household income were limited but comparable.

**Table 1.** Baseline Characteristics of People With and Without TB

	US				UK			
	People With TB		People Without TB		People With TB		People Without TB	
Characteristic	n	%	n	%	n	%	n	%
Eligible for analysis	2121	…	16 702	…	15 820	…	118 218^a^	…
Sex: male	2057	97%	16 154	97%	8145	51%	60 571	51%
Age at diagnosis: median and IQR	59	53–68	58	52–65	44	32–59	44	33–58
Race/ethnic group (known)^b^	1891	89%	15 188	91%	12 616	80%	93 402	79%
Black	745	39%	6204	41%	2059	16%	14 590	16%
White	1015	54%	7913	52%	4065	32%	32 240	35%
South Asian^c^	N/A	…	N/A	…	3972	31%	29 037	31%
Other race/ethnic group	131	13%	1071	7%	2520	20%	17 535	19%
Race/ethnic group missing	230	11%	1514	9%	3204	20%	24 816	21%
Socioeconomic measure	US: Household income				UK: Index of Multiple Deprivation			
Known/available	738	36%	10 113	61%	15 798	99%	118 070	99%
Not assigned	1383	65%	6589	39%	22	0.1%	148	0.1%
Socioeconomic measure, known^d^	738	…	10 113	…	15 798	…	118 070	…
1 (Most income/least deprived	44	6%	1233	12%	1494	9%	13 359	11%
2	142	19%	2233	22%	1766	11%	14 930	13%
3	78	11%	1455	14%	2457	16%	19 341	16%
4	359	48%	4124	40%	4078	26%	30 548	26%
5 (Least income/most deprived	115	16%	1068	11%	6003	38%	39 892	34%
Smoking status at baseline^e^								
Never	159	7%	3285	20%	8066	51%	69 688	59%
Ex-smoker	1118	53%	8866	53%	2812	18%	21 859	18%
Current smoker	742	35%	3385	20%	3755	24%	21 745	18%
Unknown	102	5%	1166	7%	1187	8%	4926	4%
BMI at baseline^e^								
<18.5 kg/m^2^	380	18%	384	2%	990	6%	3642	3%
18.5–<25 kg/m^2^	1050	50%	4491	27%	6350	40%	39 023	33%
25–<30 kg/m^2^	463	22%	5666	34%	3859	24%	32 250	27%
≥30 kg/m^2^	222	10%	5849	35%	2091	13%	20 536	17%
Unknown	6	(0.3%	312	2%	2530	16%	22 767	19%
Comorbidities and medications at baseline^f^								
COPD, asthma, or bronchiectasis	273	13%	977	6%	2198	14%	13 198	11%
Diabetes	154	7%	1391	8%	1188	8%	6695	6%
HIV-positive status	132	6%	145	1%	168	1%	251	0.2%
Hypertension	900	42%	6863	41%	2108	13%	15 276	13%
Prescribed antihypertensives^g^	821	39%	6038	36%	2450	15%	16 600	14%
Prescribed statins^g^	390	18%	3955	24%	1255	8%	8942	8%

Abbreviations: BMI, body mass index; COPD, chronic obstructive pulmonary disease; HIV, human immunodeficiency virus; IQR, interquartile range; N/A, not available; TB, tuberculosis.

^a^aUK data: 113 792 individual people without TB but due to the matching with replacement there were 118 218 total matched people without TB.

^b^Percentage of each race/ethnic group uses denominator of all “known” race/ethnic group (eg, Black US patients with TB: 745/1891 = 39%).

^c^“South Asian” included with “Other race” in US data.

^d^Percentages of quintile for socioeconomic measure use denominator of all “known” socioeconomic measure (eg, most income in US patients: 44/738 = 6%).

^e^Baseline is 720 days (∼2 years) before TB diagnosis.

^f^Mention in the medical record is taken as positive. No mention is assumed as not present.

^g^Prescribed in the 12 months before baseline (ie, prescribed 2–3 years before TB diagnosis).

Over the 4-year analysis period, among those with TB there were 462 CVD events in the US sample and 622 in the UK equating to estimated CVD incidence rates of 65 (US) and 11 (UK) per 1000 person-years (PYs). Among those without TB, 4-year CVD incidence rates were 31 (US) and 6 (UK) per 1000 PYs (Table 2).

**Table 2.** Incidence of Cardiovascular Events, Incidence Rate Ratios, and Additional Rate Ratios Adjusting for Baseline Differences

	People With TB				People Without TB						Additional Rate Ratio (95% CI) Adjusting For Differences In Baseline CVD Incidence in Pre-TB Period^b^	
	CVD Events	Person-years	Incidence per 1000 Patient-years (95% CI)		CVD Events	Person-years	Incidence per 1000 Patient-years (95% CI)		Incidence Rate Ratio (95% CI)^a^			
US data												
Pre-TB (baseline)	96	2042	47	(38–57)	565	15 833	36	(33–39)	1.1	(0.9–1.4)	1.0	…
Pre-acute	101	1464	69	(56–84)	332	11 069	30	(27–33)	1.9	(1.5–2.4)	1.7	(1.2–2.4)
Acute	117	917	127	(105–152)	209	6869	30	(26–35)	3.5	(2.7–4.4)	3.2	(2.2–4.4)
Post-acute	83	1230	67	(54–83)	244	9065	27	(24–30)	2.1	(1.6–2.7)	1.9	(1.3–2.7)
Post-TB	65	1443	45	(35–57)	289	10 406	28	(25–31)	1.3	(1.0–1.8)	1.2	(0.8–1.9)
UK data												
Pre-TB (baseline)	140	15 526	9	(8–11)	593	115 805	5	(5–6)	1.7	(1.4–2.1)	1.0	…
Pre-acute	137	11 540	12	(10–14)	474	85 762	6	(5–6)	2.1	(1.7–2.5)	1.2	(0.9–1.6)
Acute	120	7614	16	(13–19)	321	56 339	6	(5–6)	2.7	(2.2–3.3)	1.6	(1.2–2.1)
Post-acute	114	10 400	11	(9–13)	405	74 154	5	(5–6)	1.9	(1.6–2.4)	1.1	(0.9–1.5)
Post-TB	111	12 205	9	(7–11)	529	82 523	6	(6–7)	1.4	(1.1–1.8)	0.8	(0.6–1.1)
4-year totals												
US data	462	7096	65	(59–71)	1639	53 242	31	(29–32)	1.7	(1.5–2.0)	…	…
UK data	622	57 285	11	(10–12)	2322	414 584	6	(5–6)	1.9	(1.7–2.1)	…	…

Abbreviations: CI, confidence interval; CVD, cardiovascular disease; IRR, incident rate ratio; TB, tuberculosis. Pre-TB period (baseline), 1-2 years before TB diagnosis; pre-acute period, 1 year-3 m before TB diagnosis; acute period, 3 m either side of TB diagnosis; post-acute period, 3 m-1 year after TB diagnosis; post-TB period, 1-2 years after TB diagnosis.

^a^Adjusted for matching factors (sex, age, race/ethnic group, healthcare practice), socioeconomic measure, smoking, body mass index, HIV+ status, hypertension, prescribing of antihypertensives, prescribing of statins.

^b^The additional rate ratio is the ratio of 2 IRRs, taking the IRR in the pre-TB period as the baseline; 95% CIs are obtained from the full models in Stata. For example, US data acute period: IRR in acute period/IRR in pre-TB period=3.5/1.1=3.2. UK data acute period: IRR in acute period/IRR in pre-TB period=2.7/1.7=1.6.

**Figure 1.**
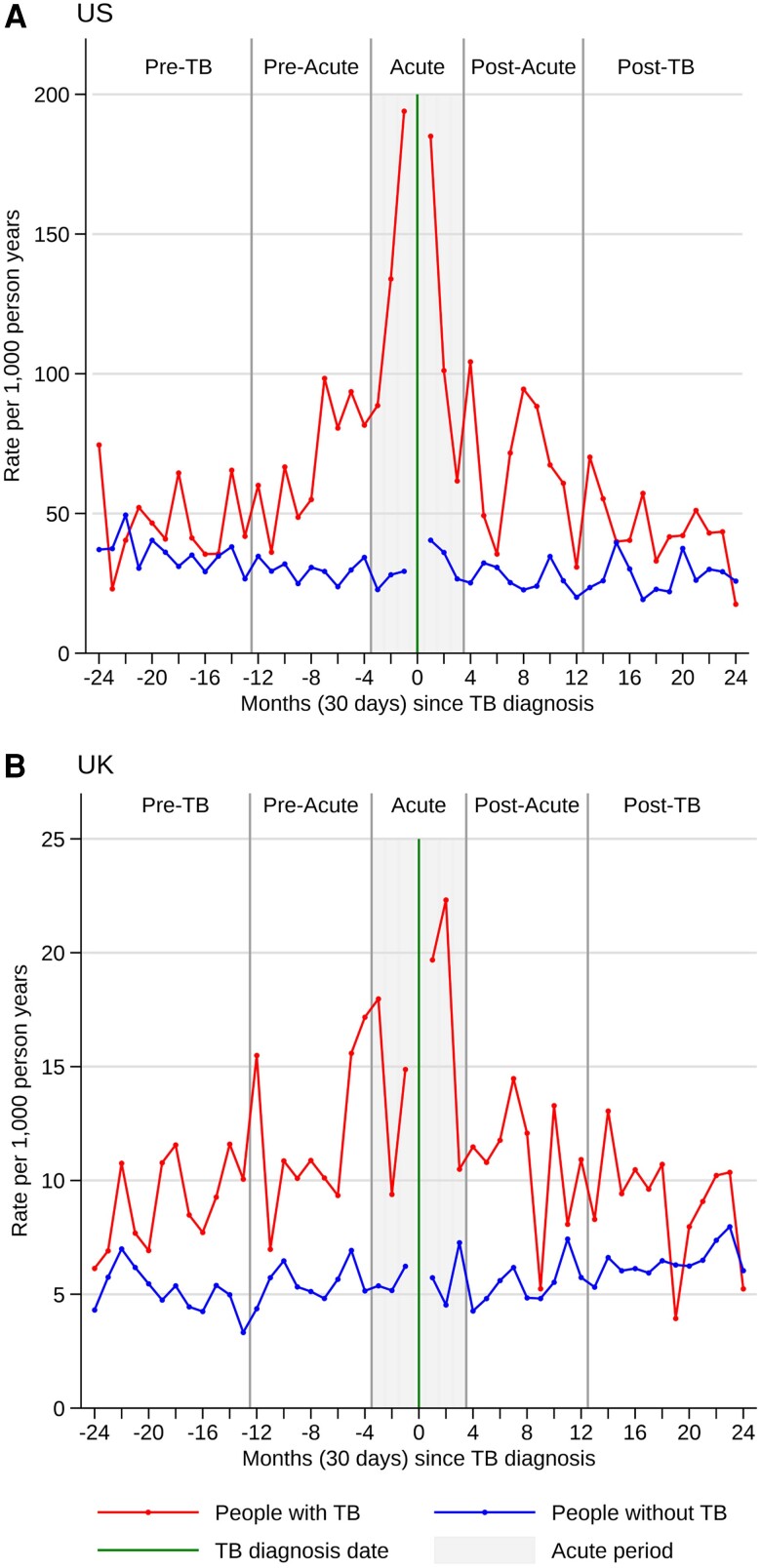
*A*, *B*, 30-day incidence rates for cardiovascular events, 2 years either side of tuberculosis diagnosis. The overall incidence rate ratio (IRR) for each period is statistically significant for US people in the preacute, acute and postacute periods and for UK people, for all periods (Table 2). Abbreviation: TB, tuberculosis.

Overall, we observed increased CVD incidence in people with TB compared to those without. People with TB had higher CVD rates during the baseline period with a marked increase in the acute period (Figure 1*A* and 1*B*). Acute period incidence among people with and without TB was 127 and 30 per 1000 PYs, respectively, in the United States; and 16 and 6 per 1000 PYs in the United Kingdom (Table 2), (Supplementary Table 1). After adjusting for covariates, the IRRs for a CVD event in people with TB in the acute period was 3.5 (95% confidence interval [CI], 2.7–4.4) in the United States and 2.7 (2.2–3.3) in the United Kingdom (compared to those without TB). After further adjusting for differences in CVD incidence in the pre-TB period, the RRs for CVD incidence during the acute period were US, 3.2 (2.2–4.4) and UK, 1.6 (1.2–2.1).

We performed stratified analyses to determine if the RRs for CVD in those with TB compared to those without differed by age, sex, or race/ethnicity (Supplementary Table 2). RRs for the acute period relative to the baseline period were similar for UK males and females. No clear patterns emerged by age or race/ethnicity. Other sensitivity analyses (Supplementary Table 3) aligned with main results.

## DISCUSSION

In this large US and UK cohort study, TB disease was associated with a significantly increased incidence of CVD before, particularly during, and after a TB diagnosis. We observed approximately 3 times increased relative incidence of CVD events during the acute period (±3 months of TB diagnosis). Importantly, we established that the higher risk of CVD incidence in the acute period persisted (RR 2–3) even after accounting for preexisting differences 1–2 years before TB diagnosis. Increased acute period CVD incidence was consistently observed in both the US and UK cohorts; this lends additional rigor and validity. Our findings have substantial implications for management of TB globally, highlighting the importance of addressing cardiovascular risk factors and care.

A key attribute of our design is that we followed people 2 years before and 2 years after (3 years in a sensitivity analysis) the date of TB diagnosis. We used 2 national healthcare databases, assembled cohorts of nearly 18 000 people with TB disease, and matched on key confounders to ∼130 000 people without TB. CVD risk was significantly higher among people with TB, even 1–2 years before their diagnosis date. Findings were similar in subanalyses limited to CVD events diagnosed in hospital, suggesting our results are not due to diagnostic bias. Our estimated RRs accounted for the preexisting differences, alleviating important concerns about potential bias because of confounding.

Observations from our study are consistent with previously published results from healthcare databases [23] but expand on existing studies and suggest a stronger relationship between TB and CVD than previously reported, particularly near the time of TB diagnosis. For example, a propensity-matched study that used US commercial insurance data reported an increased incidence of myocardial infarction within 1 year after TB diagnosis (adjusted hazard ratio, 2.0; 95% CI, 1.3–3.0) [31]. Similarly, a Taiwan insurance database study reported higher incidence of acute coronary syndrome in people with a TB history compared to non-TB control groups (2.1 vs 1.5/1000 PYs) [32].

Several mechanisms may contribute to CVD in people with TB. First, the increased CVD incidence before TB diagnosis may be attributed to LTBI-related systemic inflammation and immune activation [33, 34], incipient, or subclinical TB disease [20, 35, 36]. Pulmonary inflammation may result in vascular injury and endothelial damage [7, 37]. TB-induced adipose inflammation may also exacerbate underlying metabolic disease, influencing CVD risk [11, 38–40]. Our epidemiologic findings alongside biologically plausible mechanisms provide compelling evidence for a robust relationship between TB and CVD.

### Strengths and Limitations

Key strengths of our analyses include the large and generalizable linked cohorts with TB and CVD diagnoses. We used national VHA data from the United States and nationally representative primary care data from the United Kingdom to establish well-described cohorts of people with TB disease. Despite differences in populations and absolute CVD risk, patterns of results were comparable. Our comparison groups without TB disease were matched on age, sex, race/ethnic group, and healthcare setting, providing a robust design to adjust for potential confounding. We matched virtually all (100% US, >99% UK) to at least 1 and up to 10 people without TB disease. We used a difference-in-difference analysis, building on designs assessing CVD risk associated with acute COVID-19 [30]. This inherent adjustment for confounding resulting from fixed personal characteristics is a significant methodological advance on previous research.

Another strength was our focus on incident CVD events, reducing risk of reverse causality. We assessed the risks of CVD events at monthly intervals over an extended period, thus evaluating the timing of CVD events in relation to TB diagnosis in more detail than previous studies.

Regarding limitations, first, electronic health record diagnoses of both TB disease and CVD outcomes are imperfect. However, TB is a notifiable disease in both countries, and previous work has validated the prevalence of TB in UK primary care [41, 42]. Second, misclassification of LTBI as TB disease was possible. However, our US case definition included medications used to treat TB disease and not LTBI (ie, ethambutol and pyrazinamide). In the United Kingdom, TB medication is not prescribed through primary care; though for the low proportion (∼14%) that had prescribing data available, ethambutol and pyrazinamide were recorded, consistent with TB disease. Additionally, rates of LTBI screening are relatively low in both cohorts, particularly in the United Kingdom [43]. Third, TB diagnosis can often be delayed, and symptomatic disease may have been present for some time before our “index date,” which may thus be approximate. Diagnosis dates can also be inaccurate, but we removed people reporting a first CVD and TB diagnosis on the same day (a potential sign of data entry error) and UK participants reporting a first CVD event on 1 January 2000 (implausibly large excess on this date).

Fourth, our analysis may not have included all CVD outcomes. Although we assessed our study outcomes in both hospital and primary care data, which have been validated for both the VA and CPRD [44, 45], some CVD events may have been missed. In sensitivity analyses for the UK data, we included only CVD events that resulted in hospitalization because acute and more severe events are more accurately captured and less affected by diagnostic biases or missed documentation. Results from these analyses were consistent with our main results. A potential immortal time bias is inherent in our analysis whereby any patient who hypothetically may have been later diagnosed with TB but had an immediately fatal CVD event is not included in the pre-TB period and for this reason we did not include immediately fatal CVD events (CVD mortality events reported in mortality datasets with no prior visits to primary care or hospital with a CVD code) subsequent to the TB index date; our primary outcome thus differs slightly from the common composite outcome of major adverse cardiovascular events [46]. However, a sensitivity analysis in the UK data including these few immediately fatal events (28 in people with TB) made no difference to the RRs. Although race/ethnicity was recorded, country of birth or migration status was not available and a high proportion of TB cases occur in people not born in the United States/United Kingdom [29]; however we required a minimum of 2 years of records before TB index data so recent migrants were excluded. Finally, missing data can be a concern with electronic records, but key covariate information was highly recorded in most cases (Table 1) except SES in the United States.

Fifth, our databases were only from the United States and United Kingdom. Longitudinal studies from Low- and Middle- Income Countries (LMICs) with high TB burdens would be optimal to assess our hypotheses, but sufficiently large datasets are not available. However, the UK data included many younger individuals of South Asian or Black African Caribbean origin increasing generalizability.

Finally, we set out only to examine risk of CVD events after TB disease; other research has demonstrated higher CVD risks associated with some acute infections (eg, COVID-19, pneumonia) [30] and further work could examine whether these differ for more chronic infections such as TB.

## CONCLUSION

We found elevated risks of CVD before, especially during, and after TB diagnosis. Our findings have important clinical and research implications and indicate that CVD risk assessment during TB treatment may be warranted. A substantial proportion of TB-associated mortality may be due to CVD events, particularly among older people and those with comorbidities such as diabetes for whom deaths during TB treatment are common [47, 48]. Currently CVD risk management is seldom included in TB treatment guidelines; no specific guidance exists nationally or globally [48]. Randomized controlled trials of CVD medications have been planned but rarely carried out in people with TB; such trials seem justifiable [49].

## Supplementary Data

Supplementary materials are available at *Clinical Infectious Diseases* online. Consisting of data provided by the authors to benefit the reader, the posted materials are not copyedited and are the sole responsibility of the authors, so questions or comments should be addressed to the corresponding author.

## Supplementary Material

ciae538_Supplementary_Data

## Notes

***Author Contributions*.** Conceptualization: M. J. M., J. A. C. Funding acquisition: M. J. M., J. A. C., T. H., L. P., S. C. A., D. C., I. C., M. R. Methodology: All. Data analysis: A. K., E. L., J. A. C., I. C., M. J. M., S. d. W., D. C. Initial draft: E. L., J. A. C., M. J. M., A. K. Critical revisions to draft: all authors.

***Financial support*.** This work was supported by National Institute of Allergy and Infectious Diseases (NIAID) at the National Institutes of Health [grant number R21AI156161]. J. C. and E. L. are also supported by EDCTP funded PROTID study Preventive Treatment of Latent Tuberculosis Infection in People with Diabetes Mellitus (PROTID) study, which is part of the EDCTP2 program supported by the European Union [grant number RIA2018CO-2514-PROTID]. L. S. P. is supported in part by VHA awards CSP #2008, CSP #2002, CSP Network of Dedicated Enrollment Sites, I01 CX001899, and I01 BX005831; NIH awards R01 DK127083, R21 AI156161, and U18DP006711; and a Cystic Fibrosis Foundation award PHILLI12A0. L. S. P. is also supported in part by the Veterans Health Administration (VHA). This work is not intended to reflect the official opinion of the VHA or the US government.
