# Supplementary material for: Tuberculosis and Increased Incidence of Cardiovascular Disease: Cohort Study Using United States and United Kingdom Health Records
Source: Clin Infect Dis. 2024 Nov 4;80(2):271–9. doi: 10.1093/cid/ciae538 (PMC11848252; doi:10.1093/cid/ciae538)
Supplement: ciae538_Supplementary_Data [file ciae538_supplementary_data.pdf]

Tuberculosis and increased incidence of cardiovascular disease: Cohort study using US and UK health records

Julia A Critchley (1), Elizabeth S Limb (1), Anjali Khakharia (2), Iain M Carey (1), Sara C Auld (3,4), Stephen DeWilde (1), Tess Harris (1), Lawrence S Phillips (2,5), Derek G Cook (1), Mary K Rhee (2,5), Umar A R Chaudhry (1), Liza Bowen(1), Matthew J Magee (4)

1. Population Health Research Institute, City St George's, University of London, School of Health & Medical Sciences, Cranmer Terrace, London SW17 0RE, UK
2. Atlanta VA Health Care System, Decatur, GA, USA
3. Division of Pulmonary, Allergy, Critical Care, and Sleep Medicine, Department of Medicine, Emory School of Medicine, USA
4. Departments of Global Health and Epidemiology, Rollins School of Public Health, Emory University, 1518 Clifton Rd, NE Atlanta, GA 30322 USA
5. Division of Endocrinology and Metabolism, Department of Medicine, Emory University School of Medicine, USA

Corresponding Author:
Julia Critchley: jcritchl@sgul.ac.uk

Alternative Corresponding Author:
Matthew Magee: mjmagee@emory.edu

Supplementary Text, Figures and Tables

	Description	Pages
Supplementary Text 1	Methodological appendix	1 – 4
Supplementary Text 2	Example Stata code for the Poisson models	5
Supplementary Figure 1a	Flow chart of inclusions/exclusions for US patients	6
Supplementary Figure 1b	Flow chart of inclusions/exclusions for UK patients	7 – 8
Supplementary Table 1	Unadjusted and adjusted analyses	9
Supplementary Table 2	Stratified analyses by age, sex, race/ethnic group.	10 – 12
Supplementary Table 3	Sensitivity analyses for main outcome	13 – 14

Supplementary Text S1. Methodological Appendix

Data Sources

US data: Data on US national veteran population and their healthcare utilization (VHA) is stored within the Veterans Affairs Informatics and Computing Infrastructure (VINCI) Corporate Data Warehouse (CDW; https://tinyurl.com/2byz557c). VHA datasets were used to construct cohort information including demographics, diagnosis, laboratory measurements, pharmacy prescriptions, and outcomes. US Veterans Eligibility Trends and Statistics dataset (USVETS; https://rb.gy/ujef83) provided assessment of social determinants related to health.

UK data: The Clinical Practice Research Datalink (CPRD), a large primary care database collects anonymised patient data from a network of General Practices (Primary Care Centres)(1). More than 99% of the UK population are registered with a General Practitioner (Primary Care Physician) who provides all primary care. CPRD provides longitudinal medical records for registered patients, with 18 million individuals currently registered(1). Additionally, over 90% of CPRD practices consent to their anonymised data being individually linked to other data sources including Hospital Episode Statistics (HES - data on hospital admissions)(2), Office of National Statistics (ONS) death registration records, and the Index of Multiple Deprivation (IMD) (a geo-spatial measure providing a good proxy for SES)(3).

Race (US) / Ethnicity (UK)

In the UK, the term ethnicity is used to refer to population categories (e.g., White, South Asian, Black) which are referred to as racial categories in the US. The US also recognises ethnicity (e.g., Hispanic). To retain the within country understanding we used the terms US race/UK ethnicity throughout this manuscript, accepting that they are used in different ways in both countries.

Matching

For each person with TB, up to 10 people without TB were randomly selected. They were matched on year of birth, sex, race/ethnic group and VHA facility (US) or primary care practice (UK), creating a match-set for each person with TB. In the UK only, we chose to match with replacement to ensure that an individual with TB was not excluded due to lack of matched persons without TB. This might arise as primary care (GP) practices can be as small as 2000 patients, so to find a match for each person by age, sex and ethnic group could have been challenging. In reality, we found that only a small proportion (3.6%) of people without TB were matched to more than one person with TB. Race/ethnic information was taken from self-reported information in medical records and classified into broad race/ethnic groups for matching purposes. There were three racial groups for the US data (White, Black, Mixed/Other) and four ethnic groups for the UK data. Missing race/ethnicity was used as another category for matching (approximately 11% US and 20% UK). The proportions for ethnic group in Table 1 are based on denominators of known ethnicity.

Applying initial inclusion criteria (e.g., no prior CVD codes in their records) meant that some people with TB had to be excluded entirely, and others may have "lost" 1 or more of their matched persons without TB. To maintain the match-sets, matched people without TB were excluded if their matched person with TB had to be excluded, as were people with TB who had no remaining matched people without TB. In sensitivity analyses we repeated all analyses restricting to those with TB with at least 4 matched persons without TB and this made no difference to any of our estimates.

Cardiovascular Outcomes codes

Our overall hypothesis was that inflammation associated with TB disease might progress and destabilise underlying atherosclerosis, and thus we included all CVD codes of atherosclerotic aetiology i.e., ischemic stroke, coronary artery disease, peripheral vascular disease, transient ischaemic attack. These code lists were developed from those used in previous projects (4-7)). Our complete set of diagnostic (ICD-9 and ICD-10) code lists, including for CVD, can be seen on the SGUL figshare(8)

Immortal time bias / reason for excluding mortality outcomes

Mortality data are available for all individuals through record linkage, but only after the date of the TB diagnosis (TB index date). This is because those who died with undiagnosed TB cannot be evaluated; by definition, anyone with a CVD event who then dies before their TB diagnosis date will be unknown to us. Since we can in effect only include CVD events in those who survive and go on to have a TB diagnosis, we chose to exclude CVD events after the TB diagnosis which were only recorded as deaths and did not result in a hospital admission or primary care consultation. This avoided a type of bias known as 'immortal time bias'. In a sensitivity analysis, we included these extra CVD events subsequent to the TB index date which were only recorded in the mortality records, and this made little difference to the incidence rate ratios.

Choice of covariates in model

Co-variates were chosen a priori from those known to be associated with TB and CVD e.g., socio-economic deprivation, pre-existing co-morbidities, prescribing of statins and anti-hypertensives. Our models were additionally adjusted for these variables, but this adjustment made no difference to any of our results. This is because our analysis method used a 'difference-in-difference' model, which inherently adjusts for fixed co-variates as each person acts as their own control e.g., an individual classified as a smoker at baseline retains that classification throughout the study period, hence an estimate of their risk in the acute period adjusted for baseline risk is unaffected by their smoking status.

Statistical analysis – difference in difference method

The initial analysis plan submitted in our NIH grant application envisaged a time-to-event framework, with the date of TB diagnosis as the index date. Subsequent discussion and recognition that people with TB may be at higher risk of CVD for some period before their diagnosis date(9), and hence the importance of comparing CVD risks from before this index date and adjusting for baseline values, prompted us to employ a difference-in-difference analysis, similar to recent studies which have examined the risk of myocardial infarction for COVID-19 patients(10). This difference-in-difference analysis effectively uses those with TB "as their own controls", evaluating the risks of CVD disease longitudinally before, during and subsequent to their TB diagnosis. The benefit of this approach is that it eliminates confounding due to fixed personal characteristics during the acute TB period. This is because we fitted a group-time interaction to our models, comparing the risk of CVD events during the acute period (near the time of TB diagnosis) and subsequent to diagnosis, with a "pre-TB" period (1-2 years before TB diagnosis). The group-time interaction represents the net additional effect of acute TB, and post-TB in participants, net of the baseline CVD risk (pre-TB period) for those with TB and the comparison with their matched persons without TB (those unexposed to TB disease).

Person-time at risk was evaluated in terms of person-days of follow-up, and incident CVD events were identified in each 30-day period. Follow-up time was divided into periods of 30 days, from 2 years before the index date to 2 years after the index date which were then aggregated into five longer periods (see figure below). The effect of group (person with TB or person without TB), time

(pre-TB period: 1-2 years before date of TB index date (date of diagnosis for those with TB); pre-acute period: 1 year to 3 months before diagnosis; acute period: 3 months before to 3 months after diagnosis; post-acute period: 3 months to 1 year after diagnosis; post-TB: 1 to 2 years after diagnosis), and the group-time interaction were estimated using Poisson regression, conditioned on the match-sets with an offset for time at risk

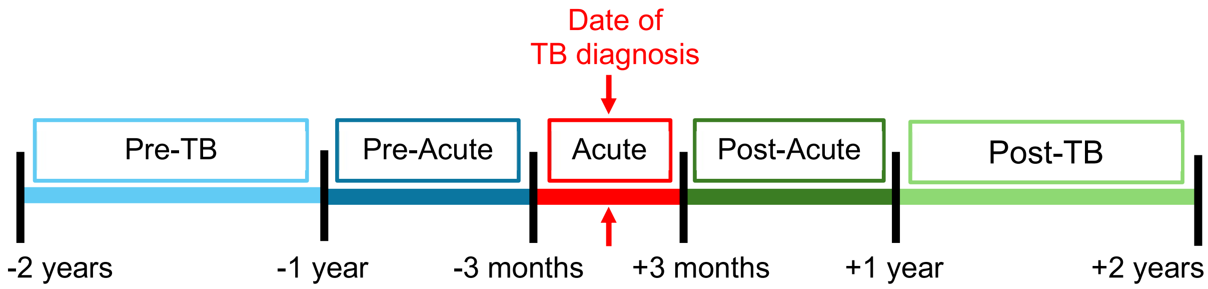

Diagnostic bias

As our analysis is based on health care records, we cannot exclude the possibility that some diagnoses of CVD were made due to increased surveillance of those undergoing TB treatment or increased consultations with a doctor before the TB diagnosis was made, a form of diagnostic bias. To reduce this risk further, in a sensitivity analysis we limited our CVD outcome to only included incident CVD diagnoses that presented in hospital (these are mainly acute myocardial infarction or stroke). The results were overall similar in this sensitivity analysis, perhaps slightly stronger. It thus seems unlikely that these acute, severe presentations of CVD would be solely due to diagnostic bias. It is not routine for UK cardiologists to test for TB disease and hence any surveillance bias in that direction is implausible.

Rationale for including two cohorts

It was a strength of our study that we were able to duplicate the same analysis in two very different datasets; a US veteran population and a population based primary care database in the UK. All Electronic Health Record databases (EHR) have limitations; in the US there were some missing data for socio-economic status for example, but this was not the case in the UK. The US has a potentially more specific case-definition (including prescribing data) because it was not possible to obtain prescribing data for TB through primary care in the UK. We thus performed our analyses in two different health record cohorts (which have different strengths and weaknesses) as a deliberate strategy to minimise the risks of bias associated with just one EHR cohort. The similar overall patterns seen are thus some reassurance that the findings cannot be explained simply by different forms of bias or missing data. There were some differences in the point estimates for the RR in the acute period, but the 95% CI almost overlap between the US and UK data.

The main models used individual-level data and due to data protection, it was not possible for the US and UK researchers to have access to each other's datasets in order to run a combined analysis.

Sensitivity analyses

Some sensitivity analyses were performed on the UK dataset alone due to resource limitations (Supplement Table 3).

1. Further adjustment for pre-existing diabetes at baseline (2 years before index date).
2. Restricting events to those in hospital data only

3. Restricting analyses to individuals with TB who had at least four matched persons without TB.
4. Including CVD events which only appeared as deaths in the mortality data i.e., a CVD event for which the individual did not have a hospital admission or primary care consultation. For reasons described above, we could only count deaths after the diagnosis of TB.
5. Adding another analysis period, 2-3 years after TB diagnosis, to look at any differences further beyond the TB diagnosis.

References

1. Wolf A, Dedman D, Campbell J, Booth H, Lunn D, Chapman J, et al. Data resource profile: Clinical Practice Research Datalink (CPRD) Aurum. Int J Epidemiol. 2019;48(6):1740-g.
2. Herbert A, Wijlaars L, Zylbersztejn A, Cromwell D, Hardelid P. Data Resource Profile: Hospital Episode Statistics Admitted Patient Care (HES APC). International Journal of Epidemiology. 2017;46(4):1093-+.
3. Mahadevan P, Harley M, Fordyce S, Hodgson S, Ghosh R, Myles P, et al. Completeness and representativeness of small area socioeconomic data linked with the UK Clinical Practice Research Datalink (CPRD). Journal of Epidemiology and Community Health. 2022;76(10):880-6.
4. Critchley JA, Carey IM, Harris T, DeWilde S, Cook DG. Variability in Glycated Hemoglobin and Risk of Poor Outcomes Among People With Type 2 Diabetes in a Large Primary Care Cohort Study. Diabetes Care. 2019;42(12):2237-46.
5. Au Yeung SL, Luo S, Schooling CM. The Impact of Glycated Hemoglobin (HbA1c) on Cardiovascular Disease Risk: A Mendelian Randomization Study Using UK Biobank. Diabetes Care. 2018;41(9):1991-7.
6. Harris T, Limb ES, Hosking F, Carey I, DeWilde S, Furness C, et al. Effect of pedometer-based walking interventions on long-term health outcomes: Prospective 4-year follow-up of two randomised controlled trials using routine primary care data. PLOS Medicine. 2019;16(6):e1002836.
7. Carey IM, Critchley JA, DeWilde S, Harris T, Hosking FJ, Cook DG. Risk of Infection in Type 1 and Type 2 Diabetes Compared With the General Population: A Matched Cohort Study. Diabetes Care. 2018;41(3):513-21.
8. Limb E, Critchley J, Carey I. Code lists for TB and CVD study. St George's, University of London. Dataset. https://doi.org/10.24376/rd.sgul.25488451.v1. 2024.
9. Magee MJ, Salindri AD, Gujral UP, Auld SC, Bao J, Haw JS, et al. Convergence of non-communicable diseases and tuberculosis: a two-way street? Int J Tuberc Lung Dis. 2018;22(11):1258-68.
10. Rezel-Potts E, Douiri A, Sun X, Chowienczyk PJ, Shah AM, Gulliford MC. Cardiometabolic outcomes up to 12 months after COVID-19 infection. A matched cohort study in the UK. PLOS Medicine. 2022;19(7):e1004052.

Supplementary Text S2. Example Stata code for the Poisson models

Adjusted model

```
1.   xtset match_id
2.   xtpoisson cv i.tb i.period io0.tb#io1.period, /*
     */   ib1.imd ib1.smoke ib2.bmig i.hiv i.hyp i.antihyp i.statin, /*
     */   re exposure(cv_time) vce(robust) irr
3.   forvalues i =1/5 {
4.        lincom 1.tb + 1.tb#`i'.period, irr
5.   }
```

1 Define **match_id** to be the panel variable. **match_id** is the ID number for each unique set of 1 person with TB and up to 10 matched people without TB.

2 xtpoisson command

`cv`	0=no CVD event; 1=CVD event
`tb`	0=person without TB; 1=person with TB
`period`	5-level variable for the 5 analysis periods<br>1=Pre-TB; 2=Pre-acute; 3=Acute; 4=Post-Acute; 5=Post-TB
`io0.tb#io1.period`	Interaction term to obtain the additional risk of CVD event in people with TB adjusting for any differences between the two TB groups at baseline (Pre-TB, period=1)
imd	Quintiles of Index of Multiple Deprivation for UK data or quintiles of household income for US data.
smoke	3-level variable for smoking plus missing<br>1=Non-smoker; 2=Ex-smoker; 3=current smoker; 0=missing
bmig	4-level variable for BMI plus missing<br>1=BMI<18.5; 2=BMI>-18.5 & <25; 3=BMI≥25 & <30; 4=BMI≥30; 0=missing
hiv	HIV+ status: 0/1 variable
hyp	Diagnosis code for hypertension: 0/1 variable
antihyp	Prescribed anti-hypertensive medication: 0/1 variable
statin	Prescribed statins: 0/1 variable
`re`	Specify random effects model
`exposure(cv_time)`	Specify **cv_time** as the time at risk. This is either time to CV event or time at risk for the 4-year analysis period.
`vce(robust)`	Specify robust standard errors
`irr`	Request exponentiated coefficients which can be interpreted as incidence rate ratios (IRR) for a Poisson model

3-5 Series of lincom commands to use linear combinations of the model estimates to obtain the IRRs for each level of the period variable.

Unadjusted model

```
xtpoisson cv i.tb i.period io0.tb#io1.period,
   */  re exposure(cv_time) vce(robust) irr
```

Supplementary Figure S1a. Flow chart showing inclusions/exclusions for US data.

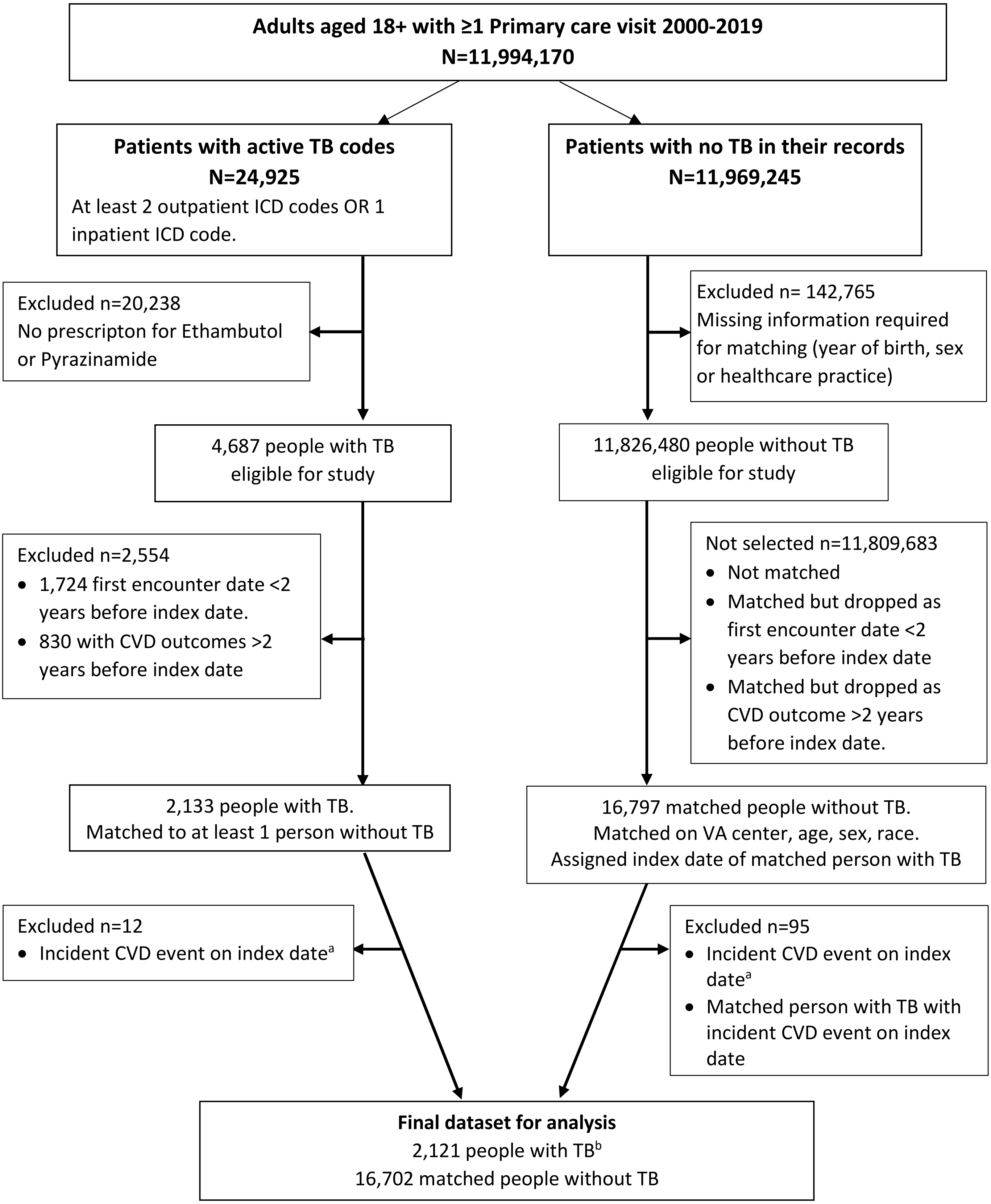

[a] People with TB who had an incident CVD event on the date of TB diagnosis were excluded as unclear if the CVD event led to the TB diagnosis. For consistency any people without TB with incident CVD event on their equivalent TB index date were also excluded.

[b] 2,034 (96%) people with TB had at least 4 matched people without TB.

Supplementary Figure S1b. Flow chart showing inclusions/exclusions for UK data

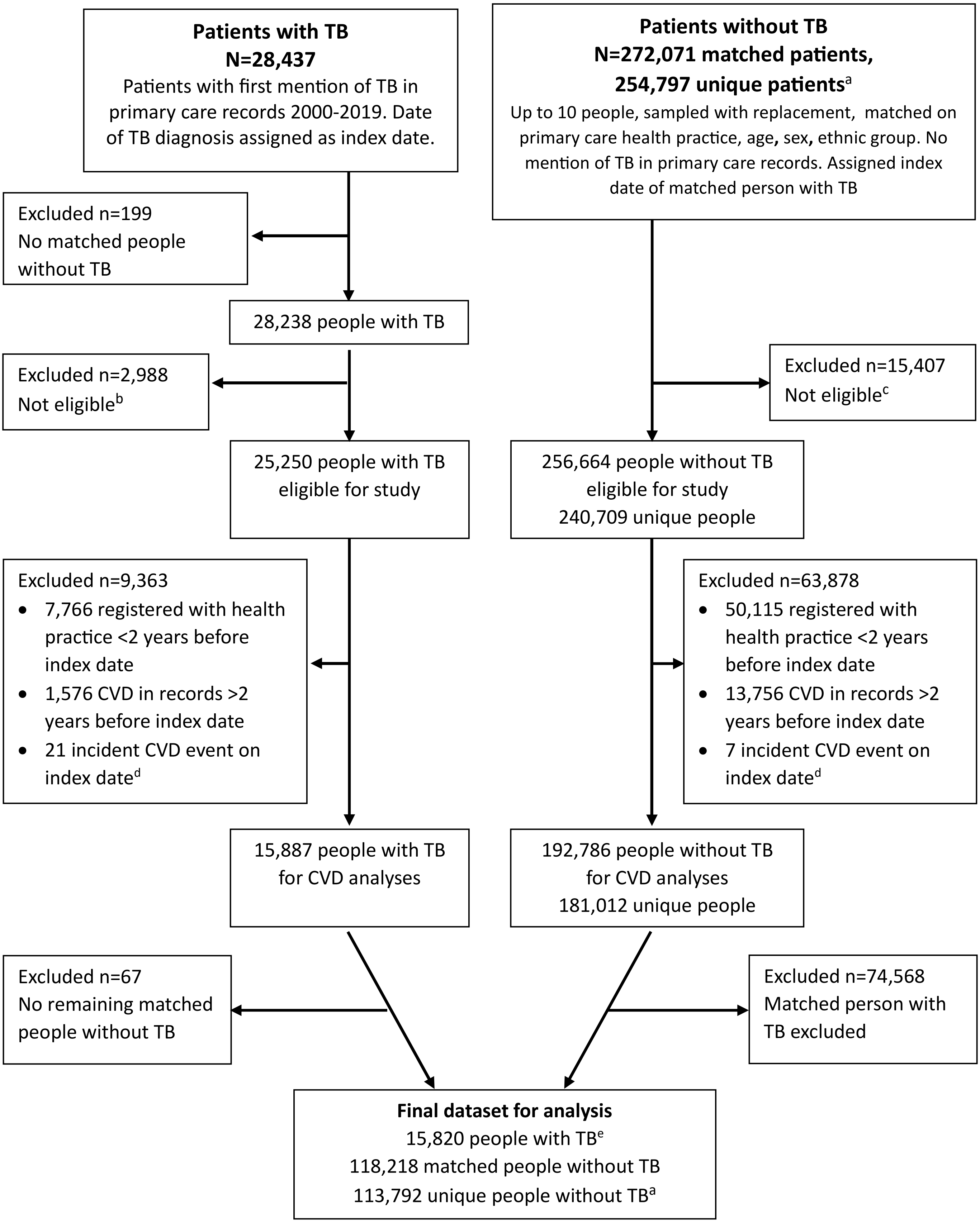

[a] Sampling with replacement was used for matching people without TB. Some people without TB were matched more than once to different people with TB.

[b] n=2,988 people with TB ineligible: 492 from primary care practices with known recording issues; 721 not eligible for hospital data linkage; 1,321 TB codes found in primary care or hospital records before index date (including TB chemoprophylaxis); 116 TB diagnosis on 01/01/2000, excluded due to possible date recording errors in CPRD data; 163 TB diagnosis in records with missing date; 131 aged 90+ at date of TB diagnosis; 44 date of death before date of TB diagnosis.

[c] n=15,407 people without TB ineligible: 4,724 from primary care practices with known recording issues; 10,056 not eligible for hospital data linkage; 188 historical TB codes found in primary care or hospital records; 439 date of death before index date.

[d] People with TB who had an incident CVD event on the TB index date were excluded as unclear if the CVD event led to the TB diagnosis. For consistency any people without TB with incident CVD event on TB index date were also excluded.

[e] 14,869 (94%) people with TB had at least 4 matched people without TB.

Supplementary Table S1. Unadjusted and adjusted analyses

	People with TB			People without TB			Adjusted for matching factors[a]		Adjusted for further covariates[b]	
	CVD events	Person years	Incidence per 1,000 person years (95% CI)	CVD events	Person years	Incidence per 1,000 person years (95% CI)	Incident rate ratio (95% CI)	Additional rate ratio (95% CI)[c]	Incident rate ratio (95% CI)	Additional rate ratio (95% CI)[c]
**US data**										
Pre-TB	96	2,042	47 (38, 57)	565	15,833	36 (33, 39)	1.3 (1.0, 1.7)	1.0	1.1 (0.9, 1.4)	1.0
Pre-acute	101	1,464	69 (56, 84)	332	11,069	30 (27, 33)	2.2 (1.6, 3.1)	1.7 (1.2, 2.4)	1.9 (1.5, 2.4)	1.7 (1.2, 2.4)
Acute	117	917	127 (105, 152	209	6,869	30 (26, 35)	4.0 (2.7, 5.9)	3.1 (2.2, 4.4)	3.5 (2.7, 4.4)	3.2 (2.2, 4.4)
Post-acute	83	1,230	67 (54, 83)	244	9,065	27 (24, 30)	2.4 (1.5, 3.7)	1.9 (1.3, 2.7)	2.1 (1.6, 2.7)	1.9 (1.3, 2.7)
Post-TB	65	1,443	45 (35, 57)	289	10,406	28 (25, 31)	1.5 (0.9, 2.6)	1.2 (0.8, 1.9)	1.3 (1.0, 1.8)	1.2 (0.8, 1.9)
**UK data**										
Pre-TB	140	15,526	9 (8, 11)	593	115,805	5 (5, 6)	1.7 (1.4, 2.1)	1.0	1.7 (1.4, 2.1)	1.0
Pre-acute	137	11,540	12 (10, 14)	474	85,762	6 (5, 6)	2.1 (1.7, 2.6)	1.2 (0.9, 1.6)	2.1 (1.7, 2.5)	1.2 (0.9, 1.6)
Acute	120	7,614	16 (13, 19)	321	56,339	6 (5, 6)	2.7 (2.1, 3.4)	1.5 (1.2, 2.1)	2.7 (2.2, 3.3)	1.6 (1.2, 2.1)
Post-acute	114	10,400	11 (9, 13)	405	74,154	5 (5, 6)	1.9 (1.5, 2.5)	1.1 (0.8, 1.5)	1.9 (1.6, 2.4)	1.1 (0.9, 1.5)
Post-TB	111	12,205	9 (7, 11)	529	82,523	6 (6, 7)	1.3 (1.0, 1.8)	0.8 (0.6, 1.0)	1.4 (1.1, 1.8)	0.8 (0.6, 1.1)

Pre-TB period (baseline), 1-2 years before TB diagnosis; Pre-acute period, 1 year-3 months before TB diagnosis; Acute period, 3 months either side of TB diagnosis; Post-acute period, 3 months-1 year after TB diagnosis; Post-TB, 1-2 years after TB diagnosis.

[a] Adjusted for matching factors: age, sex, race/ethnic group and healthcare center.

[b] Adjusted for socio-economic measure, smoking, BMI, HIV+ status, hypertension, prescribing of anti-hypertensives, prescribing of statins.

[c] The additional rate ratio is the ratio of two IRRs, taking the IRR in the Pre-TB period as the baseline, 95% CIs are obtained from the full models in Stata.

US data, unadjusted, acute period: IRR in acute period / IRR in pre-TB period = 4.0 / 1.3 = 3.1

US data, adjusted, acute period: IRR in acute period / IRR in pre-TB period = 3.5 / 1.1 = 3.2

Supplementary Table S2. Stratified analyses by sex, age-group and race/ethnic group

		People with TB		People without TB			
		CVD events	Incidence per 1,000 person years (95% CI)	CVD events	Incidence per 1,000 person years (95% CI)	Adjusted for matching factors (95% CI)[a]	Additional rate ratio (95% CI)[b]
**US data**							
Pre-TB	All subjects	96	47 (38, 57)	565	36 (33, 39)	1.3 (1.0, 1.7)	1.0
Pre-acute	All subjects	101	69 (56, 84)	332	30 (27, 33)	2.2 (1.6, 3.1)	1.7 (1.2, 2.4)
**Acute**	**All subjects**	**117**	**127 (105, 152)**	**209**	**30 (26, 35)**	**4.0 (2.7, 5.9)**	3.1 (2.2, 4.4)
Post-acute	All subjects	83	67 (54, 83)	244	27 (24, 30)	2.4 (1.5, 3.7)	1.9 (1.3, 2.7)
Post-TB	All subjects	65	45 (35, 57)	289	28 (25, 31)	1.5 (0.9, 2.6)	1.2 (0.8, 1.9)
**UK data**							
Pre-TB	All subjects	140	9 (8, 11)	593	5 (5, 6)	1.7 (1.4, 2.1)	1.0
Pre-acute	All subjects	137	12 (10, 14)	474	6 (5, 6)	2.1 (1.7, 2.6)	1.2 (0.9, 1.6)
**Acute**	**All subjects**	**120**	**16 (13, 19)**	**321**	**6 (5, 6)**	**2.7 (2.1, 3.4)**	1.5 (1.2, 2.1)
Post-acute	All subjects	114	11 (9, 13)	405	5 (5, 6)	1.9 (1.5, 2.5)	1.1 (0.8, 1.5)
Post-TB	All subjects	110	9 (7, 11)	529	6 (6, 7)	1.3 (1.0, 1.8)	0.8 (0.6, 1.0)
**Stratified by sex, males and females**							
**US data (males only due to small numbers in females)**							
Pre-TB	Males	95	48 (39, 58)	563	37 (34, 40)	1.3 (1.0, 1.7)	1.0
Pre-acute	Males	98	69 (56, 84)	328	31 (27, 34)	2.2 (1.5, 3.1)	1.7 (1.2, 2.4)
**Acute**	**Males**	**115**	**129 (107, 155)**	**207**	**31 (27, 36)**	**4.0 (2.7, 5.8)**	3.1 (2.2, 4.4)
Post-acute	Males	82	69 (55, 85)	243	28 (24, 31)	2.4 (1.5, 3.7)	1.9 (1.3, 2.7)
Post-TB	Males	62	44 (34, 57)	282	28 (25, 32)	1.5 (0.9, 2.5	1.2 (0.7, 1.8)
**UK data**							
Pre-TB	Males	87	11 (9, 13)	356	6 (5, 7)	1.8 (1.4, 2.3)	1.0
Pre-acute	Males	86	14 (12, 18)	289	7 (6, 7)	2.1 (1.6, 2.9)	1.2 (0.9, 1.7)
**Acute**	**Males**	**75**	**19 (15, 24)**	**205**	**7 (6, 8)**	**2.6 (1.8, 3.6)**	1.5 (1.0, 2.1)
Post-acute	Males	62	12 (9, 15)	221	6 (5, 7)	1.9 (1.3, 2.7)	1.1 (0.7, 1.6)
Post-TB	Males	68	11 (9, 14)	307	7 (7, 8)	1.4 (1.0, 2.1)	0.8 (0.5, 1.2)
Pre-TB	Females	53	7 (5, 9)	237	4 (4, 5)	1.7 (1.2, 2.2)	1.0
Pre-acute	Females	51	9 (7, 12)	185	4 (4, 5)	2.0 (1.4, 2.8	1.2 (0.8, 1.9)
**Acute**	**Females**	**45**	**12 (9, 16)**	**116**	**4 (3, 5)**	**2.8 (1.9, 4.1)**	1.7 (1.1, 2.7)
Post-acute	Females	52	10 (8, 13)	184	5 (4, 6)	2.0 (1.4, 2.8)	1.2 (0.8, 1.8)
Post-TB	Females	42	7 (5, 9)	222	5 (5, 6)	1.2 (0.8, 1.9)	0.7 (0.5, 1.2)

		People with TB		People without TB			
		CVD events	Incidence per 1,000 person years (95% CI)	CVD events	Incidence per 1,000 person years (95% CI)	Adjusted for matching factors (95% CI)[a]	Additional rate ratio (95% CI)[b]
**Stratified by age, <60 and 60+**							
**US data**							
Pre-TB	<60	37	34 (24, 47)	190	20 (17, 23)	1.7 (1.2, 2.5	1.0
Pre-acute	<60	34	43 (30, 61	141	21 (17, 24)	2.1 (1.4, 3.1)	1.2 (0.7, 2.1)
**Acute**	**<60**	**39**	**77 (55, 106)**	**89**	**20 (16, 25)**	**3.7 (2.3, 5.9)**	2.2 (1.3, 3.8)
Post-acute	<60	37	53 (37, 73)	122	21 (17, 25)	2.5 (1.5, 4.1)	1.5 (0.8, 2.6)
Post-TB	<60	26	31 (20, 45)	163	23 (20, 27)	1.3 (0.7, 2.4)	0.8 (0.4, 1.5)
Pre-TB	60+	59	61 (46, 79)	375	59 (53, 65)	1.0 (0.8, 1.4)	1.0
Pre-acute	60+	67	98 (76, 124)	191	45 (38, 51)	2.2 (1.5, 3.1)	2.1 (1.4, 3.2)
**Acute**	**60+**	**78**	**188 (149, 235)**	**120**	**47 (39, 56)**	**3.9 (2.7, 5.8)**	3.8 (2.4, 6.0)
Post-acute	60+	46	86 (63, 115)	122	39 (32, 46)	2.2 (1.3, 3.5)	2.1 (1.2, 3.6)
Post-TB	60+	39	65 (46, 89)	126	37 (31, 44)	1.7 (1.0, 3.1)	1.7 (0.9, 3.1)
**UK data**							
Pre-TB	<60	39	3 (2, 4)	182	2 (2, 2)	1.6 (1.2, 2.3)	1.0
Pre-acute	<60	35	4 (3, 5)	141	2 (2, 2)	1.9 (1.3, 2.7)	1.2 (0.7, 1.9)
**Acute**	**<60**	**44**	**7 (5, 10)**	**99**	**2 (2, 3)**	**3.4 (2.3, 4.8)**	2.0 (1.2, 3.4)
Post-acute	<60	46	6 (4, 8)	121	2 (2, 2)	2.8 (2.0, 3.9)	1.7 (1.0, 2.8)
Post-TB	<60	45	5 (3, 6)	201	3 (3, 3)	1.5 (1.1, 2.1)	0.9 (0.6, 1.5)
Pre-TB	60+	101	27 (22, 33)	411	16 (15, 18)	1.7 (1.3, 2.1)	1.0
Pre-acute	60+	102	38 (31, 46)	333	18 (16, 20)	2.1 (1.6, 2.7)	1.2 (0.9, 1.7)
**Acute**	**60+**	**76**	**44 (35, 55)**	**222**	**19 (17, 22)**	**2.3 (1.6, 3.2)**	1.4 (1.0, 1.9)
Post-acute	60+	68	30 (23, 38)	284	19 (17, 21)	1.5 (1.1, 2.2)	0.9 (0.6, 1.3)
Post-TB	60+	65	25 (19, 32)	328	20 (18, 23)	1.2 (0.8, 1.8)	0.7 (0.5, 1.1)
**Stratified by race/ethnic group, white and non-white**							
**US data**							
Pre-TB	White	49	50 (37, 66)	317	42 (38, 47)	1.2 (0.8, 1.7)	1.0
Pre-acute	White	59	85 (65, 109)	177	34 (29, 40)	2.4 (1.5, 3.8)	2.1 (1.3, 3.2)
**Acute**	**White**	**63**	**146 (112, 187)**	**117**	**37 (30, 44)**	**3.8 (2.3, 6.2)**	3.3 (2.0, 5.3)
Post-acute	White	49	85 (63, 113)	124	30 (25, 35)	2.7 (1.5, 4.9)	2.4 (1.4, 4.0)
Post-TB	White	32	48 (33, 68)	159	34 (29, 39)	1.3 (0.7, 2.7)	1.2 (0.6, 2.1)

		People with TB		People without TB			
		CVD events	Incidence per 1,000 person years (95% CI)	CVD events	Incidence per 1,000 person years (95% CI)	Adjusted for matching factors (95% CI)[a]	Additional rate ratio (95% CI)[b]
Pre-TB	Non-white	36	42 (30, 58)	189	27 (23, 31)	1.5 (1.0, 2.4)	1.0
Pre-acute	Non-white	36	59 (41, 81)	121	25 (20, 29)	2.4 (1.4, 3.9)	1.5 (0.9, 2.6)
**Acute**	**Non-white**	**39**	**101 (71, 137)**	**74**	**24 (19, 30)**	**4.1 (2.2, 7.6)**	2.7 (1.5, 4.7)
Post-acute	Non-white	26	49 (32, 72)	101	24 (20, 29)	2.0 (1.0, 3.9)	1.3 (0.7, 2.4)
Post-TB	Non-white	20	31 (19, 49)	109	22 (18, 27)	1.4 (0.6, 3.4)	0.9 (0.4, 2.0)
**UK data**							
Pre-TB	White	50	13 (9, 16)	238	8 (7, 9)	1.6 (1.2, 2.3)	1.0
Pre-acute	White	52	18 (13, 23)	203	9 (8, 10)	2.0 (1.3, 2.8)	1.2 (0.8, 1.9)
**Acute**	**White**	**36**	**18 (13, 26)**	**135**	**9 (7, 10)**	**2.0 (1.3, 3.1)**	1.2 (0.8, 2.0)
Post-acute	White	32	12 (8, 17)	152	7 (6, 9)	1.6 (1.0, 2.5)	1.0 (0.6, 1.6)
Post-TB	White	48	15 (11, 20)	238	10 (9, 11)	1.4 (0.9, 2.4)	0.9 (0.5, 1.4)
Pre-TB	Non-white	49	6 (4, 8)	205	3 (3, 4)	1.7 (1.2, 2.3)	1.0
Pre-acute	Non-white	52	8 (6, 11)	161	4 (3, 4)	2.3 (1.6, 3.2)	1.3 (0.9, 2.1)
**Acute**	**Non-white**	**48**	**12 (8, 15)**	**101**	**3 (3, 4)**	**3.3 (2.2, 4.9)**	2.0 (1.2, 3.1)
Post-acute	Non-white	56	10 (7, 13)	145	4 (3, 4)	2.6 (1.8, 3.7)	1.5 (1.0, 2.4)
Post-TB	Non-white	41	6 (4, 8)	170	4 (3, 4)	1.5 (1.0, 2.3)	0.9 (0.6, 1.5)

Pre-TB period (baseline), 1-2 years before TB diagnosis; Pre-acute period, 1 year-3 months before TB diagnosis; Acute period, 3 months either side of TB diagnosis; Post-acute period, 3 months-1 year after TB diagnosis; Post-TB, 1-2 years after TB diagnosis.

[a] Models for stratified analyses were adjusted for matching factors only (age, sex, race/ethnic group, healthcare center) as the stratified models adjusted for further covariates failed to converge for the US data. In the 'all subjects' analysis, the additional rate ratios were almost identical for models adjusted for matching factors and models adjusted for further covariate (Supp Table 1).

[b] The additional rate ratio is the ratio of two IRRs, taking the IRR in the Pre-TB phase as the baseline. Estimates and 95% CIs are obtained from the full models in Stata.

US data, acute period: IRR in acute period / IRR in pre-TB period = 4.0 / 1.3 = 3.1

Supplementary Table S3. Sensitivity analyses for UK data

	People with TB			People without TB				
	**CVD events**	**Person years**	**Incidence per 1,000 person years (95% CI)**	**CVD events**	**Person years**	**Incidence per 1,000 person years (95% CI)**	**Incident rate ratio (95% CI)[a]**	**Additional rate ratio (95% CI) adjusting for differences in baseline CVD incidence in Pre-TB period**
**Main analysis, CVD events in primary care or hospital data [a]**								
Pre-TB	140	15,526	9 (8, 11)	593	115,805	5 (5, 6)	1.7 (1.4, 2.1	1.0
Pre-acute	137	11,540	12 (10, 14)	474	85,762	6 (5, 6)	2.1 (1.7, 2.5)	1.2 (0.9, 1.6)
**Acute**	**120**	**7,614**	16 (13, 19)	**321**	**56,339**	6 (5, 6)	2.7 (2.2, 3.3)	1.6 (1.2, 2.1)
Post-acute	114	10,400	11 (9, 13)	405	74,154	5 (5, 6)	1.9 (1.6, 2.4)	1.1 (0.9, 1.5)
Post-TB	110	12,205	9 (7, 11)	529	82,523	6 (6, 7)	1.4 (1.1, 1.8)	0.8 (0.6, 1.1)
**1. Further adjustment for diabetes diagnosis at baseline**								
Pre-TB	140	15,526	9 (8, 11)	593	115,805	5 (5, 6)	1.7 (1.4, 2.0)	1.0
Pre-acute	137	11,540	12 (10, 14)	474	85,762	6 (5, 6)	2.0 (1.7, 2.5)	1.2 (0.9, 1.6)
**Acute**	**120**	**7,614**	**16 (13, 19)**	**321**	**56,339**	6 **(5, 6)**	2.6 (2.1, 3.2)	1.6 (1.2, 2.1)
Post-acute	114	10,400	11 (9, 13)	405	74,154	5 (5, 6)	1.9 (1.5, 2.4)	1.1 (0.9, 1.5)
Post-TB	110	12,205	9 (7, 11)	529	82,523	6 (6, 7)	1.4 (1.1, 1.7)	0.8 (0.6, 1.1)
**2. CVD events from hospital data only**								
Pre-TB	66	15,529	4 (3, 5)	293	115,816	3 (2, 3)	1.6 (1.3, 2.1)	1.0
Pre-acute	60	11,543	5 (4, 7)	257	85,771	3 (3, 3)	1.7 (1.3, 2.2)	1.0 (0.7, 1.5)
**Acute**	**63**	**7,616**	**8 (6, 11)**	**158**	**56,346**	**3 (2, 3)**	**2.9 (2.1, 3.9)**	1.8 (1.2, 2.6)
Post-acute	55	10,402	5 (4, 7)	216	74,161	3 (3, 3)	1.8 (1.3, 2.4)	1.1 (0.7, 1.6)
Post-TB	63	12,206	5 (4, 7)	277	82,534	3 (3, 4)	1.5 (1.1, 2.0)	0.9 (0.6, 1.4)
**3. Restricting to persons with TB who had at least 4 matched people without TB**								
Pre-TB	131	14,595	9 (7, 11)	579	113,742	5 (5, 6)	1.7 (1.4, 2.1)	1.0
Pre-acute	126	10,851	12 (10, 14)	459	84,244	5 (5, 6)	2.1 (1.7, 2.5)	1.2 (0.9, 1.6)
**Acute**	**118**	**7,160**	**16 (14, 20)**	**315**	**55,342**	**6 (5, 6)**	**2.8 (2.3, 3.5)**	1.6 (1.2, 2.2)
Post-acute	106	9,780	11 (9, 13)	400	72,842	5 (5, 6)	1.9 (1.5, 2.4)	1.1 (0.8, 1.5)
Post-TB	105	11,476	9 (7, 11)	515	81,065	6 (6, 7)	1.4 (1.1, 1.8)	0.8 (0.6, 1.1)

	People with TB			People without TB				
	CVD events	Person years	Incidence per 1,000 person years (95% CI)	CVD events	Person years	Incidence per 1,000 person years (95% CI)	Incident rate ratio (95% CI)[a]	Additional rate ratio (95% CI) adjusting for differences in baseline CVD incidence in Pre-TB period
**4. Including additional CVD events only recorded as deaths and with no mention of CVD in primary care or hospital records[b]**								
Pre-TB	140	15,526	9 (8, 11)	593	115,805	5 (5, 6)	1.7 (1.4, 2.1)	1.0
Pre-acute	137	11,540	12 (10, 14)	474	85,762	6 (5, 6)	2.1 (1.7, 2.5)	1.2 (0.9, 1.6)
**Acute**	**127**	**7,614**	**17 (14, 20)**	**332**	**56,339**	**6 (5, 7)**	**2.7 (2.2, 3.4)**	1.6 (1.2, 2.1)
Post-acute	127	10,400	12 (10, 14)	434	74,154	6 (5, 6)	2.0 (1.6, 2.5)	1.2 (0.9, 1.6)
Post-TB	118	12,205	10 (8, 12)	560	82,523	7 (6, 7)	1.4 (1.1, 1.7)	0.8 (0.6, 1.1)
**5. Extending follow-up to 3 years after TB diagnosis[c]**								
Pre-TB	140	15,526	9 (8, 11)	593	115,805	5 (5, 6)	1.7 (1.4, 2.1)	1.0
Pre-acute	137	11,540	12 (10, 14)	474	85,762	6 (5, 6)	2.1 (1.7, 2.6)	1.2 (0.9, 1.6)
**Acute**	**120**	**7,614**	**16 (13, 19)**	**321**	**56,339**	**6 (5, 6)**	**2.7 (2.2, 3.4)**	1.6 (1.2, 2.1)
Post-acute	114	10,400	11 (9, 13)	405	74,154	5 (5, 6)	2.0 (1.6, 2.5)	1.1 (0.9, 1.5)
Post-TB	111	12,205	9 (7, 11)	529	82,523	6 (6, 7)	1.4 (1.1, 1.8)	0.8 (0.6, 1.1)
Post TB 2-3	100	10,579	9 (8, 11)	416	68,071	6 (6, 7)	1.5 (1.1, 2.0)	0.9 (0.6, 1.2)

Pre-TB period (baseline) is 1-2 years before TB diagnosis; pre-acute period is 1 year-3 months before TB diagnosis; Acute period is 3 months either side of TB diagnosis; Post acute period is 3 months-1 year after TB diagnosis; post-TB period is 1-2 years after TB diagnosis.

[a] Adjusted for matching factors (age, sex, race/ethnic group, healthcare center), socio-economic measure, smoking, BMI, HIV+ status, hypertension, prescribing of anti-hypertensives, prescribing of statins.

[b] Sensitivity analysis 4 only includes those additional CVD events occurring as deaths occurring **after** the TB diagnosis. Any individual who died before receiving a diagnosis of TB are unknown to us.

[c] Sensitivity analysis 5 has an additional period 'Post-TB 2-3' which is 2-3 years after TB diagnosis.
